# New horizons in clinical practice guidelines for use with older people

**DOI:** 10.1093/ageing/afae158

**Published:** 2024-07-24

**Authors:** Finbarr C Martin, Terence J Quinn, Sharon E Straus, Sonia Anand, Nathalie van der Velde, Rowan H Harwood

**Affiliations:** Population Health Sciences, Faculty of Life Sciences and Medicine, King’s College London, London, UK; School of Cardiovascular and Metabolic Health, College of Medical, Veterinary and Life Sciences, University of Glasgow, Glasgow, UK; Department of Medicine, University of Toronto and Li Ka Shing Knowledge Institute of St. Michael’s, Toronto, Ontario, Canada; Departments of Medicine and Health Research Methods, Evidence, and Impact, Faculty of Health Sciences, McMaster University, Hamilton, Ontario, Canada; Department of Internal Medicine, Section of Geriatric Medicine, University of Amsterdam, Amsterdam, The Netherlands; Amsterdam Public Health Research Institute (Aging and Later Life), Amsterdam, The Netherlands; School of Health Sciences, University of Nottingham, Nottingham, UK

**Keywords:** older people, guidelines, methodology, applicability, evidence-based, patient-centred

## Abstract

Globally, more people are living into advanced old age, with age-associated frailty, disability and multimorbidity. Achieving equity for all ages necessitates adapting healthcare systems. Clinical practice guidelines (CPGs) have an important place in adapting evidence-based medicine and clinical care to reflect these changing needs. CPGs can facilitate better and more systematic care for older people. But they can also present a challenge to patient-centred care and shared decision-making when clinical and/or socioeconomic heterogeneity or personal priorities are not reflected in recommendations or in their application. Indeed, evidence is often lacking to enable this variability to be reflected in guidance. Evidence is more likely to be lacking about some sections of the population. Many older adults are at the intersection of many factors associated with exclusion from traditional clinical evidence sources with higher incidence of multimorbidity and disability compounded by poorer healthcare access and ultimately worse outcomes. We describe these challenges and illustrate how they can adversely affect CPG scope, the evidence available and its summation, the content of CPG recommendations and their patient-centred implementation. In all of this, we take older adults as our focus, but much of what we say will be applicable to other marginalised groups. Then, using the established process of formulating a CPG as a framework, we consider how these challenges can be mitigated, with particular attention to applicability and implementation. We consider why CPG recommendations on the same clinical areas may be inconsistent and describe approaches to ensuring that CPGs remain up to date.

## Key points

- Evidence is comparatively sparse about people living with frailty, multimorbidity or cognitive impairment or marginalised groups.
- New methodological approaches can mitigate but not remove the impact of evidence gaps.
- Recommendations should take into account variations in priorities to support patient-centred care.
- Older adults and stakeholders with expertise from experience should be included at all stages of clinical practice guideline (CPG) development.
- Evidence-based methods to support implementation can be included as supplementary guidance to CPG recommendations.

## Introduction

Most healthcare systems share the challenge of adapting their services and clinical practices to better meet the needs of the growing numbers of older people, more of whom are living into advanced old age, with age-associated frailty, multimorbidity and disability. Frailty and multimorbidity contribute to marked clinical heterogeneity among older adults and present a challenge to clinicians. Outcomes, including benefits, risks and experiential burdens of even the more straightforward interventions, such as prescribed medications, become less predictable. Moreover, the value placed on the potential clinical gains versus the risks and burdens differs between individuals. For example, more emphasis may be placed on function or comfort than survival.

Developments in medical technologies present clinicians with the challenge of staying up to date. As clinical practice becomes more complex, clinicians use authoritative guidance such as clinical practice guidelines (CPGs) to both direct and justify their practice. Practising evidence-based medicine requires ‘integrating individual clinical expertise with the best available external clinical evidence from systematic research’ [1], and CPGs are taken to represent this best evidence [2]. Therefore, the suitability of CPGs to guide clinical practice is a key requirement if healthcare can successfully adapt to meet the needs of older patients.

Application of high-quality CPGs has improved clinical outcomes and experience of older patients in clinical areas such as hip fracture [3] and stroke [4]. Conversely, CPGs may be of limited use if they do not reflect the clinical needs and priorities of the intended beneficiaries, and uncritical application of CPGs may lead to ‘too much medicine’, leading to iatrogenic harm [5]. It was highlighted previously that the evidence available and the CPG recommendations formulated from them did not meet requirements for many older patients in several high-income countries [6–8]. For example, only 1 of 17 guidelines approved by the Australian National Health and Medical Research Council (NHMRC) considered multimorbidity [6].

**Figure 1:**
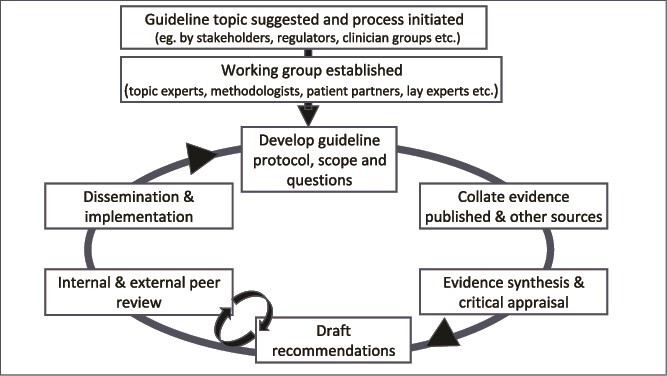
Process of creating a clinical practice guideline. Note the iterative nature of the process. Suggested for further reading: https://academic.oup.com/intqhc/article/28/1/122/2363781.

Frameworks and standards for CPG development have been described [9–11]. (see Figure 1) In addition, van Munster *et al.* have proposed enhancing the focus on older people to improve applicability [12]. Huang *et al.* suggested that a key task of the United Nations Decade of Health Ageing was to update CPGs relevant to older people in three respects: increasing the number and diversity of older participants in primary research; development of evidence synthesis approaches that maximise the useful knowledge from research, for example, by identifying the impact of clinical and demographic heterogeneity; and to improve implementation with new tools to support shared decision-making, e.g. clinical vignettes [13].

The aim of this paper is to discuss the challenges of developing and implementing CPGs fit for the ageing clinical populations, by which we mean CPGs that can be feasibly applied to support equitable, effective and person-centred healthcare for older adults.

## Heterogeneity, evidence and clinical practice guidelines

Ageing is associated with pharmacological and physiological changes that impact therapeutic benefits, harms and burdens of treatment. Older populations are particularly heterogenous, variable lifelong influences being reflected in various degrees of multimorbidity, frailty and disability. The 2019 Global Burden of Diseases report listed the commonest causes of loss of disability-adjusted life years, in this order: ischaemic heart disease, stroke, chronic obstructive pulmonary disease (COPD), dementias and diabetes [14].

Most CPGs for chronic conditions focus on one disease [5], but considering any of these in isolation is likely to be inadequate as adults living with two or more long-term conditions outnumber those living with one, and multimorbidity incidence increases with age [15]. Age alone is a limited guide to heterogeneity, as socioeconomic factors strongly impact age of onset of multimorbidity [16].

Frailty is associated with worse outcomes from many therapeutic interventions [17] and medication-related harms, independent of polypharmacy [18]. Frailty, multimorbidity and associated treatments therefore affect the balance of therapeutic benefits and harms. Evidence-based treatment for one condition may exacerbate another condition. Efficacy of medications differ due to the presence of a comorbidity affecting access or adherence, drug absorption, kinetics, or end organ effects. Coincident treatment may interact. Therefore, when people with multimorbidity are receiving multiple medications, the benefits, burdens and harms are less predictable [19]. These considerations should impact judicious use of CPGs, and considering them throughout CPG development potentially adds evidence to support shared decision-making.

## Clinical practice guideline scope and governance

The scope of guidance recommendations may define what is considered important for clinical services or individual encounters. Most CPG development panels include content and methodologic experts, but to enhance person-centred healthcare, CPGs must harness the contribution of people and their essential care partners so that their priorities and values are reflected in each stage of guideline development [20]. Patient and caregiver involvement influences CPG scope with inclusion of patient-relevant topics, outcome selection, approaches to recommendation development and implementation [21]. Panels need to work with those representative sections of the older population in whom the condition of interest is highly prevalent and particularly those whose experience of the clinical area is adversely affected by inequities [22]. Equity should be considered from determining scope and conception of the questions through data abstraction, analysis and formulating recommendations [23]. As yet, women, racialised people, particularly racialised women and those with lower socioeconomic resources are underrepresented in guideline panels [24, 25].

Complete transparency and mitigation of possible bias arising from potential conflicts of interest (COI) should be mandated in all CPGs [26].

## Seeking the evidence

The CPG scope and resulting questions or topic areas inform the broad evidence search. This is perhaps the stage at which the greatest thought must be given to the intended benefits of a CPG and where the greatest challenges arise in making CPGs relevant for older people (Table 1). It must focus not only on what evidence is needed but also on its applicability. Bias in research is not necessarily by methodological weakness, but more subtle by the framing and focus of the primary research questions, the outcomes selected and selective reporting of results [27, 28].

**Table 1:** Challenges to the use of CPGs with older people

• Lack of relevant primary evidence on treatment efficacy on important subgroups • Need to take account of multimorbidity and to avoid polypharmacy • Limitation in CPG scope, evidence and synthesis and how evidence is translated to usable recommendations • Heterogeneity of the population—clinical, socioeconomic and cultural, challenges validity and appropriateness of recommendations • Variation in priorities and preferences between individuals and requirement for shared decision-making • Misapplication and poor implementation

### Are clinical trials representative?

The traditional ‘building blocks’ of therapy-focused CPGs are randomised controlled trials (RCTs). For pharmacological treatments, there has been some improvement but still inadequate inclusion of older people in clinical trials. A ‘geriatrics search filter’ has been proposed to improve access to data on older participants in relevant research reports [29]. However, people living in more socioeconomically deprived neighbourhoods are less likely to be included in clinical trials [30]. For example, recruitment from a primary care population or 8902 patients was analysed according to cardiovascular risk level, ethnicity and socio-economic deprivation: those at higher cardiovascular risk were underrepresented, specifically those in higher deprivation areas and those identifying as black African or Caribbean ethnicity [heritage] [31]. Little progress has been made in reflecting the heterogeneity represented by frailty or functional ability in the consideration of benefits, harm and burdens [32, 33], so evaluations frequently fail to include those at highest risk of adverse health events or those with the most to gain, such as from COVID vaccination [34].

Using neighbourhood-level indicators of social determinants of health has been suggested as a way to identify and purposively address this during recruitment to broaden the generalisability of clinical trials [30]. In practice, recruiting disadvantaged or marginalised populations to trials is difficult, and new approaches are required to trial design and recruitment, or different data sources will have to be used.

### Identifying and addressing inequity

Tackling health inequities requires consideration of the differential impacts of healthcare, which may arise from interventions being less accessible, less effective or more burdensome for some groups.

Absence of data on identifiable subgroups may compromise the broad applicability of CPG recommendations. For example, a systematic review of rehabilitation interventions for patients after a hip fracture demonstrated that over a quarter of studies excluded participants based on equity factors, notably cognitive impairment or residence in care homes [35]. Thus, factors to consider include both the range of diversity in the primary data and data analysis exploring differential uptake, adherence, adverse effects or outcomes according to subgroups, especially if this can be done using individual participant data across multiple studies. Crafting the search questions or extraction approaches to explore potential associations may generate different knowledge [36] and enable recommendations framed to reduce inequity [22]. The PROGRESS-Plus framework offers a systematic approach to identifying factors relating to inequity in research [37, 38]. This granularity may enhance the utility of evidence for formulating CPG recommendations and highlight the nature of further research needed [39]. A limitation of many primary evidence sources is that these characteristics are treated as independent confounders or covariates (thereby assuming that they are irrelevant to describing a treatment effect). This approach fails to consider that individuals’ vulnerability characteristics are ‘multiple and intersecting’ [40].

### Complex interventions

Many useful clinical treatments addressing multifaceted conditions such as geriatric syndromes are complex interventions, comprising multiple interacting components acting across several domains and requiring behaviour change by many players in a multidisciplinary environment. They usually require adaptation to different implementation contexts [41], which may not be easily identified and are not necessarily generalisable to other healthcare settings. The contribution of context and comparison with ‘usual care’ is difficult to interpret unless systematically described by use of TiDieR (template for intervention description and replication) guidelines [42].

### New evidence sources

Data from randomised controlled trials may therefore not adequately correspond to the scope or address the questions posed by patients and clinical practice. Data collected from routine service delivery, especially ‘big data’ from electronic records, or a bespoke clinical cohort can provide ‘real-world evidence’ (RWE) and may have a role in informing CPGs [43, 44]. Such data are prone to biases and confounding, so they are relatively weak in determining treatment effects, and they are more often used in studying adverse effects. An example using routinely collected primary care data enabled stratification of falls risk in older people starting antihypertensive treatment [45]. However, RWE data offer better population representativeness and, due to their massive size, sufficient power to analyses subgroups.

In anticipation of such use, consideration should be given to the routine collection of socioeconomic, demographic and functional ability descriptors, to add to the RWE base for sections of the population underrepresented in RCTs. This evidence can complement or be an alternative to data from RCTs where this is absent. The National Institute for Health and Care Excellence (NICE) produced a detailed framework for the use of RWE. Central messages are around the need for high-quality source data, transparent reporting and use of sensitivity and bias analyses to test the robustness of conclusions [46]. Although RWE is useful, we should not stop advocating for more research specific to older adults and designed to enable participation of the widest range of older people in terms of health status and ethnographic characteristics.

In practice, CPG recommendations may be supported by a variety of evidence types. For example, the upcoming Scottish Intercollegiate Guidelines Network (SIGN) guideline on dementia care drew the most appropriate cognitive assessment from a test accuracy paradigm; the sections on distress involved a synthesis of evidence from various complex trial designs, and evidence in the section describing grief was predominantly based on qualitative data [47].

### Which outcomes matter?

Well-conducted trials may be lacking in important evidence if outcomes do not capture the issues of importance to patients, such as functional ability. CPGs can be enhanced if clinical trials include relevant patient-reported outcomes such as quality of life, functional status, cognitive functioning, hospitalisation and treatment burden in addition to the usual morbidity/mortality outcomes [12, 48].

Orthodox clinical trials insist on the specification of a single primary outcome, largely to defend against type 1 statistical error (a ‘false-positive’ trial). Complex interventions, where context has an important bearing on outcomes, and with multiple stakeholders, are likely to need consideration of multiple outcomes. For example, an intervention may have effects on disease occurrence, impairments, functional ability, mood and quality of life among participants, but for application in practice, data on carer impact, e.g. caregiver stress [49] or health service outcomes, are needed. Multiple secondary outcomes are often reported from trials, but better standardisation is needed to enable synthesis such as by meta-analysis. Time to benefit is also an important consideration when CPGs may be applied for people with limited life expectancy. Pragmatic trials that evaluate whether interventions work in routine clinical settings should be included in searches to enable an assessment of feasibility to inform recommendations [50].

## Evidence synthesis and triangulation

Evidence does not speak for itself. There is inevitably a need for appraisal and interpretation. The increasing complexity of clinical questions requiring CPG recommendation is matched by increasing sophistication in methods for evidence synthesis, and there is a growing array of powerful methods to collate evidence to inform guidelines [51, 52]. Meta-analysis aims to combine the findings of multiple studies, thereby increasing precision (better estimates of treatment effects) and allowing for exploration of heterogeneity between studies. Given that primary trials in older adults are likely to be complex, methods for meta-analysis using data from cluster designs [53], qualitative studies [54] and exploring indirect comparisons through networks are especially relevant [55, 56]. Evidence synthesis from observational studies may be useful to generate hypotheses if they highlight both beneficial and adverse events in clinical populations under-represented in individual studies.

Noting the assessment of individual study bias as part of the systematic review process is important when deciding how to summate the evidence before incorporation into recommendations [57]. Publication bias, for example, omission of ‘negative’ studies, imprecision in results and study quality are important limitations to the confident grading of recommendations.

The need for inclusive diversity in the primary research and at the evidence extraction and synthesis stage is two-fold. Firstly, representativeness of the research participants means that recommendations may be applicable to an undifferentiated target population for treatment. Secondly, to enable differentiation in analyses by measured characteristics that could then be clinically applied either by modifying recommendations or by alerting the clinicians to appraise the applicability of the recommendations when implementing with their patient. An approach to differentiate four groups was proposed by van Muster *et al*. among older people: (i) relatively healthy older people; (ii) older people with one additional specific (interfering) comorbid condition; (iii) older people with multimorbidity; and (iv) older people with known vulnerabilities such as falls, delirium or functional limitations [12]. Exploring treatment effects in subgroups, for example, to investigate equity associated differences may be feasible [58], but grouping individuals into social demographic, racial or ethnic categories is problematic and controversial, particularly if the purpose is to create groups for comparisons. The objections arise from the statistical challenges [59] and the rationale underpinning the distinct groups [60]. Race and ethnicity are social constructs without evidence to support differential biology that might have pathophysiological significance. The application of small but statistically significant between-group differences to guide individual clinical decisions is scientifically invalid if the groups defined lack biological plausibility relevant to the condition or treatment [60, 61].

## Evidence to recommendations

Recommendation formulation requires a critical approach to the research findings, noting the precision of effect estimates derived from evidence synthesis, the limitations and potential biases. Publication bias, for example, omission of ‘negative’ studies, imprecision in results and study quality are important limitations to the confident grading of recommendations. Qualitative data are also subject to various selection and information biases.

Consideration of health inequities may inform the weighing of evidence, the recommendation and implementation guidance [22, 23]. A systematic approach is needed to achieving consensus on the applicability of findings to different contexts and population groups and incorporating the values and preferences of the intended beneficiaries [11, 62].

Heterogeneity of context and practical implementation of complex interventions in RCTs adds uncertainty about burden and outcome predictions and thus formulating recommendations that can be feasibly or effectively implemented. Decisions may be based on factors that are difficult to measure and correct for. Even with sophisticated statistical adjustments, there will always be concerns around confounding when making inferences. This can result in an inconclusive evidence summation and a ‘no recommendation’ in the CPG. Collating multiple evidence types may help, despite caveats about data from RWE and cohort observational studies. When a range of the different types and quality of data consistently support the same conclusion, this may be sufficient to make practice recommendations. This process of triangulation is the basis for drawing conclusions in newer methodologies such as realist synthesis, which may also include qualitative data [63].

As ageing-associated changes impact the balance of benefit/harm/burdens of treatment, it may seem sensible to apply age-specific guidance as has been done by NICE for secondary fracture prevention, but as chronological age is less discriminating within the older population, it is clinically more credible to incorporate other factors such as comorbidities, frailty, functional ability and cognitive function, as has been suggested for treatment target levels for hypertension [64]. A consensus-based set of recommendations has been proposed to support CPG developers to make recommendations more applicable to multimorbid patient populations. The recommendations cover all the stages up to formulating recommendations and grading their strength [5].

Many of the systems used for describing strength of CPG recommendations also ask for consideration of ‘indirectness’, i.e. does the included evidence relate to the populations, interventions or outcomes of interest [10]. For CPGs with an older adult focus, recommendations that rely on extrapolation from trials in middle-aged participants are necessarily downgraded on this characteristic. It may be that no evidence-based recommendation specific to older adults can be made. In the absence of empirical data, there is still value in collating clinical experience and wisdom, and some guidelines complement evidence-based recommendations with expert opinion statements [65]. These are often based on consensus across a broad multidisciplinary group, and guidance on the conduct and reporting of such consensus opinion exercises is available [66].

**Figure 2:**
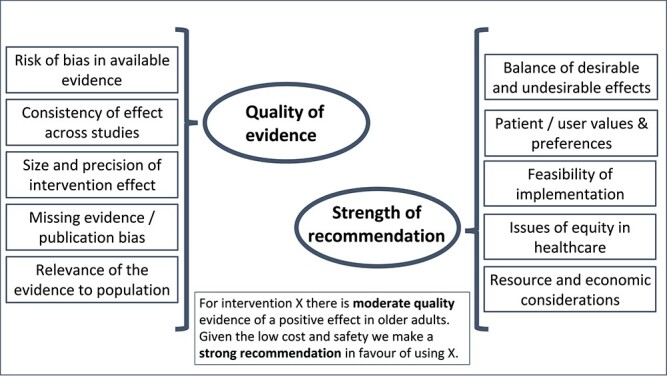
Considerations in formulating a guideline recommendation. Factors that influence the quality of evidence and the strength of recommendation are distinct but complementary and combine to give a standard text. Further reading: https://www.gradeworkinggroup.org/.

Whatever approaches are taken to address the challenges described above, the CPG should explain the mechanism used for upgrading or downgrading levels of evidence into recommendations [67] (Figure 2). External peer review of draft recommendations with relevant experts or stakeholders is recommended [68]. Inclusion of the public and patients in this review varies and the optimal approach is not yet clear [69].

### Inconsistencies in clinical practice guidelines

Differences between CPG recommendations may reflect the distinct uses that the sponsors or guideline panel have in mind. This is illustrated by osteoporosis treatment guidelines for which recommendations from the National Osteoporosis Guidelines Group UK (NOGG) [70], which integrate available evidence on clinical efficacy, effectiveness and safety, differ from those of NICE, which applies a cost-effectiveness threshold for drug treatments [71]. Differences may also arise from variable applicability to specific patient groups, for example, treatment guidelines for hypertension [72], where both treatment thresholds and drug choices differ.

## Implementation

Guidelines are only helpful if recommendations are implementable and implemented. AGREE-II guidance and the AGREE-REX tool offer a systematic approach to implementation guidance [73]. Barriers and facilitators may exist at the level of individuals (e.g. patients, clinicians), organisation (e.g. hospital, community) and healthcare system. Evidence on effective implementation strategies can potentially inform evidence to recommendations decisions, but this depends on greater consistency in describing strategies [74] and their impact [75]. The evidence may suggest who will need additional support, such as financing, transport, language and training caregivers.

### Patient-centred application of clinical practice guidelines

For individual clinical decisions, the guideline recommendation must be appraised at three levels [76]. Is the supporting evidence valid? Does it apply to this clinical situation? Does it apply to this person’s particular circumstances, priorities and values? Practice standardisation without flexibility threatens patient-centred decision-making. The California Healthcare Foundation/American Geriatrics Society guidelines for the care of older patients with diabetes incorporated ‘time to benefit’ in its guidance for long-term preventive therapy [77]. Guidance can incorporate shared decision-making to support implementation, as in the Canadian Task Force on Preventive Care fragility fracture prevention guidance [78]. As part of the SHARE-IT project, Heen *et al.* have developed decision aids using vignettes to be used alongside evidence summaries and recommendations to support shared decision-making [79].

### Adapting to resource settings

Suitability of recommendations dependent upon evidence drawn from populations in high-income countries may or may not enhance health outcomes or reduce inequity for people living elsewhere. High-level evidence on older people living in low- and middle-income countries is scant. It may be possible to tailor recommendations to resource implications [80]. The 2022 World Falls Guidelines provided separate prevention interventions geared to low- and-middle income countries, albeit with limited supporting evidence [81].

## Sustainability of clinical practice guidelines

The explosion in availability of biomedical science brings challenges not generally matched by agility in guideline production [82]. Using AI or crowdsourcing to identify new research may help [83]. Developments in study designs and evidence synthesis will hopefully generate more granular knowledge to inform updated CPGs better reflecting the heterogeneity of older patients. ‘Living guidelines’ may help to systematically update recommendations when sufficient new evidence emerges, according to predefined criteria [84], as illustrated by the Australian Stroke Foundation’s Guideline motivated by the rapidly evolving stroke evidence base [85].

Rapid reviews offer an accelerated approach to meeting urgent or changing population health needs [86] as exemplified by three produced over 8 months to address issues facing care homes staff and residents during the COVID-19 pandemic [87] and a rapid review of hospital-at-home-style ‘Virtual Wards’ [49].

## Conclusion

**Figure 3:**
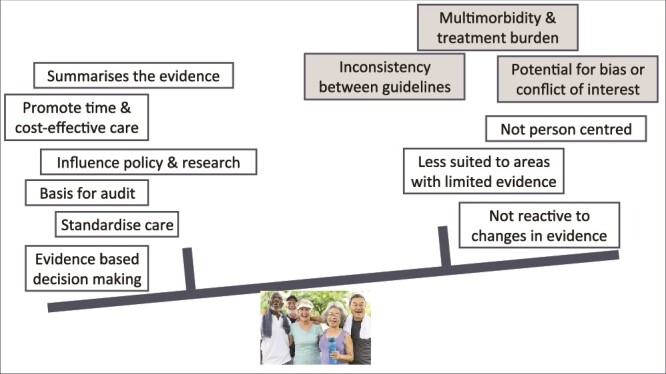
Advantages and disadvantages of clinical practice guidelines for older people. Note the fine balance between benefits and harms, additional factors such as conflict of interest or multiplicity of guidelines could tip the balance unfavourably. Note that some of negative factors (dark shaded) can be overcome, others not so. Further reading: https://www.sciencedirect.com/science/article/pii/S0020138322000778.

CPGs will likely continue to have a prominent place in healthcare. Guidance on how to recognise trustworthy guidelines has been provided [88], but clinicians need also to remember that applicability may be limited by what is researched, on which participants, what evidence is extracted and what factors and which stakeholders have been considered when formulating recommendations. Clinicians must therefore apply guidelines thoughtfully, ensuring that patient characteristics and context do not compromise their applicability or lead to unintended adverse consequences. The developments in treatments, with greater complexity characterising the most effective responses to geriatric syndromes in older people, and changes in how healthcare is provided can adversely affect the delivery of patient-centred care. Use of CPGs for older people has both benefits and risks (Figure 3). Methodological developments can mitigate some of these challenges, but others require a broader mindset, with a focus on utilising a broader set of evidence sources and addressing health inequities. Involving older people and marginalised sections of the population will be necessary to achieve this.
